# IQ Domain-Containing GTPase-Activating Protein 1 Regulates Cytoskeletal Reorganization and Facilitates NKG2D-Mediated Mechanistic Target of Rapamycin Complex 1 Activation and Cytokine Gene Translation in Natural Killer Cells

**DOI:** 10.3389/fimmu.2018.01168

**Published:** 2018-05-28

**Authors:** Alex M. Abel, Aradhana A. Tiwari, Zachary J. Gerbec, Jason R. Siebert, Chao Yang, Nathan J. Schloemer, Kate J. Dixon, Monica S. Thakar, Subramaniam Malarkannan

**Affiliations:** ^1^Department of Microbiology and Immunology, Medical College of Wisconsin, Milwaukee, WI, United States; ^2^Laboratory of Molecular Immunology and Immunotherapy, Blood Research Institute, Milwaukee, WI, United States; ^3^Department of Pediatrics, Medical College of Wisconsin, Milwaukee, WI, United States; ^4^Department of Medicine, Medical College of Wisconsin, Milwaukee, WI, United States

**Keywords:** IQ domain-containing GTPase-activating protein 1, natural killer cells, cytoskeleton, NKG2D, IFN-γ, translation, mechanistic target of rapamycin, ribosomal protein S6

## Abstract

Natural killer (NK) cells are innate lymphocytes that play essential roles in mediating antitumor immunity. NK cells respond to various inflammatory stimuli including cytokines and stress-induced cellular ligands which activate germline-encoded activation receptors (NKRs), such as NKG2D. The signaling molecules activated downstream of NKRs are well defined; however, the mechanisms that regulate these pathways are not fully understood. IQ domain-containing GTPase-activating protein 1 (IQGAP1) is a ubiquitously expressed scaffold protein. It regulates diverse cellular signaling programs in various physiological contexts, including immune cell activation and function. Therefore, we sought to investigate the role of IQGAP1 in NK cells. Development and maturation of NK cells from mice lacking IQGAP1 (*Iqgap1^−/−^*) were mostly intact; however, the absolute number of splenic NK cells was significantly reduced. Phenotypic and functional characterization revealed a significant reduction in the egression of NK cells from the bone marrow of *Iqagp1^−/−^* mice altering their peripheral homeostasis. Lack of IQGAP1 resulted in reduced NK cell motility and their ability to mediate antitumor immunity *in vivo*. Activation of *Iqgap1^−/−^* NK cells *via* NKRs, including NKG2D, resulted in significantly reduced levels of inflammatory cytokines compared with wild-type (WT). This reduction in *Iqgap1^−/−^* NK cells is neither due to an impaired membrane proximal signaling nor a defect in gene transcription. The levels of *Ifng* transcripts were comparable between WT and *Iqgap1^−/−^*, suggesting that IQGAP1-dependent regulation of cytokine production is regulated by a post-transcriptional mechanism. To this end, *Iqgap1^−/−^* NK cells failed to fully induce S6 phosphorylation and showed significantly reduced protein translation following NKG2D-mediated activation, revealing a previously undefined regulatory function of IQGAP1 *via* the mechanistic target of rapamycin complex 1. Together, these results implicate IQGAP1 as an essential scaffold for NK cell homeostasis and function and provide novel mechanistic insights to the post-transcriptional regulation of inflammatory cytokine production.

## Introduction

Natural killer (NK) cells are innate lymphocytes that mediate many immune functions including antiviral immunity and tumor clearance (1). NK cell activation occurs in response to cytokines as well as stress-induced ligands present on transformed or infected cells (2). These ligands activate NK cells through germline-encoded receptors (NKRs) and, along with inhibitory receptors that primarily interact with MHC class I, specify the NK cell activation repertoire (3). NK cells acquire these receptors at various stages throughout their development (4); however, unlike lymphocytes that mediate adaptive immunity, NK cells do not require either a clonotypic receptor or prior antigen-specific sensitization to become fully activated (5). In response to tumor ligands, the NK cell response can be separated into two primary effector functions. First, NK cells are cytotoxic lymphocytes and can directly lyse transformed cells. Second, NK cells promote the antitumor immune response through the secretion of inflammatory cytokines.

Although these functions often occur concurrently in response to NKR-mediated stimulation, they are regulated through distinct signaling mechanisms, and the molecules involved in these processes are subject to regulation at multiple levels (6–8). Scaffold proteins are well-known molecular rheostats capable of fine-tuning NK cell signaling in various circumstances (9). For instance, the cytohesin-associated scaffolding protein (CASP) regulates intracellular trafficking and facilitates cell migration as well as IFN-γ secretion in NK cells (10). Another example includes kinase suppressor of Ras (KSR1), a mitogen-activated protein kinase (MAPK) scaffold, which is required for NK cell lytic activity (11). Similarly, a study using the NK-like cell line, YTS, demonstrated a role for IQ domain-containing GTPase-activating protein 1 (IQGAP1) in mediating antitumor cytotoxicity (12). Given the complex nature and incomplete understanding of how scaffold proteins regulate NK cell biology, we seek to understand further the molecular mechanisms that govern NK cell function through further investigation of the multifunctional scaffold protein, IQGAP1.

IQ domain-containing GTPase-activating protein 1 is a ubiquitously expressed, 190-kDa scaffold protein (13) that regulates cellular signaling in multiple contexts (9, 14, 15). The scaffold function of IQGAP1 is mediated through six functional domains that facilitate its interaction with various proteins and signaling molecules. These interactions enable IQGAP1 to regulate and myriad of signaling pathways critical for diverse cellular functions. Specifically, IQGAP1 has been shown to regulate cytoskeletal reorganization (16, 17) as well as multiple signaling pathways activated downstream of receptor tyrosine kinases and G protein-coupled receptors. These include estrogen receptor (18), calmodulin (19) MAPK (20), Akt (14, 21), mechanistic target of rapamycin (mTOR) (22), and phosphoinositide (23) signaling pathways. Interestingly, the role of IQGAP1 can differ based on physiological context. For instance, IQGAP1 regulates Erk1/2 phosphorylation in mouse embryonic fibroblasts (24) and RAS-driven tumors (25); however, MAPK signaling occurs independently of IQGAP1 in other cancer cell lines (26).

Therefore, it is not surprising that IQGAP1 regulates immune cell function through diverse mechanisms. For example, IQGAP1 facilitates phagocytosis (27, 28) and caspase-1 activation (29) in macrophages. In neutrophils, IQGAP1 mediates cell migration by promoting CXCR2 signaling processes (30). In B cells, IQGAP1 participates in B cell receptor signaling at the immunological synapse by regulating microtubule organizing center (MTOC) polarization (31); By contrast, IQGAP1 acts as a negative regulator of T cell receptor signaling (32) and nuclear factor of activated T cell-mediated transcription in T cells (33). Given the multitude of IQGAP1-regulated functions, it is expected that IQGAP1 facilitates multiple signaling processes within a specific population of immune cells and, although one study has described a role for IQGAP1 in regulating NK cell cytotoxicity (12), it is likely that IQGAP1 facilitates other NK cell functions.

Regulation of cellular metabolism has been widely studied in lymphocytes (34) and is often directly regulated by the kinase, mTOR (35). The role of mTOR has recently been appreciated in regulating IL-15 signaling, and it is essential for the development and function of NK cells (36, 37). Specifically, mechanistic target of rapamycin complex 1 (mTORC1)-induced metabolic reprogramming is prerequisite for NK cell activation (37, 38), and mTORC1 activation is required for NK cell effector functions in response to NKR stimulation (39). The components of mTORC1 include the central kinase, mTOR, proline-rich Akt substrate 40, mammalian lethal with Sec13 protein 8 (mLST8), DEP-domain-containing mTOR-interacting protein (Deptor), and, the defining component of mTORC1, a regulatory-associated protein of mTOR (Raptor) (40). Many cellular processes, such as cell growth, cell division, autophagy, and protein translation, are regulated by mTORC1 (41), which reflects its role as a critical regulator of cellular physiology. IQGAP1 is among many proteins that interact with mTORC1 (42). Of note, this interaction is highly conserved as mTORC1 controls cell division in yeast *via* Iqg1p, the yeast homolog of IQGAP1 (43). In mammalian cells, the interaction between IQGAP1 and mTORC1 promotes proliferation of fibroblasts (44) and hepatocellular carcinoma cell lines (45). Furthermore, the bacterial effector protein, OspB, regulates mTORC1 activity in an IQGAP1-dependent manner to promote cell proliferation during *Shigella* infection (46).

IQ domain-containing GTPase-activating protein 1 regulates wide range of cellular processes critical for lymphocyte function; therefore, we sought to (1) investigate the role of IQGAP1 in mediating NK cell homeostasis and effector functions and (2) describe potential mechanisms by which IQGAP1 facilitates cytoskeletal reorganization and NKR activation in the context of NK cell signaling and function. Using a global IQGAP1 knockout mouse (*Iqgap1^−/−^*), we observed defects in cytoskeletal reorganization in NK cells lacking IQGAP1. These alterations were associated with reduced bone marrow (BM) egress and *in vitro* motility as well as decreased tumor clearance *in vivo*. *Iqgap1^−/−^* NK cells also showed impaired post-transcriptional cytokine production in response to NKG2D stimulation. This observation was associated with decreased NKG2D-induced mTORC1 activation and global protein synthesis. Our results demonstrate multiple roles for IQGAP1 in facilitating NK cell function and define a novel mechanism in which IQGAP1 positively regulates mTORC1 activation to facilitate cytokine translation in NK cells.

## Experimental Procedures

### Mice and Tumor Cell Lines

*Iqgap1^−/−^* mice were generously provided by our collaborator, André Bernards (Massachusetts General Hospital, Center for Cancer Research, Charlestown, MA, USA). These mice were of a mixed genetic background consisting of C57BL/6 and 129/SJL. Therefore, we bred these mice back to C57BL/6 mice for 11 generations resulting in C57BL/6 *Iqgap1^−/−^* mice. The wild-type (WT) (control mice) used in this study were generated from the same *Iqgap1^+/−^* × *Iqgap1^+/−^* breeding used to generate the F11-C57BL6 *Iqgap1^−/−^* mice. All mice were maintained in pathogen-free conditions at the Biological Resource Center at the Medical College of Wisconsin (MCW), Milwaukee, WI, USA. All animal protocols were approved by the institutional IACUC committee. EL4, RMA, RMA/S, and YAC-1 cell lines were purchased from ATCC (Rockville, MD, USA) and maintained in RPMI-1640 medium containing 10% heat-inactivated FBS (Life Technologies, Grand Island, NY, USA). Generation of *H60*-expressing EL4 stable cell lines has been described (47).

### Flow Cytometry

Single-cell preparations from BM, spleen, or IL-2-cultured NK cells were stained and analyzed as previously described (47), and NK cells were gated as either CD3ε^−^NK1.1^+^ or CD3ε^−^NCR1^+^ as indicated in the figure legends. Flow cytometry was performed using LSR-II (BD Biosciences, San Jose, CA, USA) or MACSQuant Analyzer 10 (Miltenyi Biotec, Bergisch Gladbach, Germany) cytometers. Data analysis was completed using the FlowJo software (FlowJo, Ashland, OR, USA). Fluorochrome-conjugated antibodies for the following markers were used for evaluation of cell surface expression: NK1.1 (PK136), CD3ε (17A2), CD27 (LG.7F9), CD11b (M1/70), KLRG1 (2F1), Ly49A (A1), Ly49G2 (4D11), Ly49D (4E5), Ly49H (3D10), NKG2D (A10), NCR1 (29A1.4), CD49b (DX5), and CD98 (RL388) were purchased from eBioscience (San Diego, CA, USA) and Ly49C/I (5E6; BD Pharmingen). Live/dead analysis was done using the FITC Annexin V Apoptosis Detection Kit (BD Biosciences) according to the manufacturer’s instructions. For *in vivo* labeling of NK cells, fluorochrome-conjugated anti-CD45.2 (104) was purchased from eBioscience (San Diego, CA, USA) and 2 µg of anti-CD45.2 was injected i.v. (retro-orbital) per mouse. Intracellular cytokine and chemokine quantification was completed using fluorochrome-labeled IFN-γ (XMG1.2) and CCL3 (DNT3CC) antibodies purchased from eBioscience (San Diego, CA, USA). The intracellular staining procedure was done as previously described (48). Intracellular phospho-protein analysis using fluorochrome-conjugated p-S6 Ser240/244 (D68F8) was completed according to the manufacturer’s instructions (Cell Signaling Technologies, Beverly, MA, USA). Briefly, IL-2 cultured NK cells were activated for 1 h with anti-NKG2D mitogenic antibody before being fixed in 100% ice-cold methanol, permeabilized, and incubated with p-S6 at 1:300 for 1 h. Cells were then washed, surface stained with anti-NK1.1, and analyzed on the MACSQuant Analyzer 10 cytometer.

### NK Cell Isolation and Culture

Isolation of splenic NK cells was done using negative selection with the EasySep™ Mouse NK Cell Isolation Kit according to the manufacturer’s instructions (STEMCELL Technologies, Vancouver, BC, Canada). Isolated NK cells were determined to be >85% (CD3ε^−^NK1.1^+^). Generation of IL-2-cultured NK cells was previously described (49). Briefly, single-cell suspensions from spleen and BM were passed through nylon wool columns to deplete adherent populations consisting of B cells and macrophages. Nylon wool-non-adherent cells were cultured with 1,000 U/ml of IL-2 (NCI-BRB-Preclinical Repository, Maryland, MD, USA). The purity of the NK cultures was checked, and preparations with more than 90% of NK1.1^+^ cells were used on day 7–10. Experiments were conducted with IL-2-cultured NK cells unless otherwise specified.

### Confocal Microscopy and Live Cell Imaging

Confocal analyses were performed in Olympus FluoView FV1000 MPE microscope that is equipped with multiphoton capabilities (MaiTai DSBB-OL, 710–990 nm; MaiTai DSHP-OL, 690–1,040 nm). NK cells were plated in poly-l-lysine-coated (MilliporeSigma) LabTak chamber slides (Thermo Fisher Scientific, Waltham, MA, USA) for staining and imaging. Cells were fixed/permeabilized with 4% paraformaldehyde for 15 min and blocked for 1 h with 3% BSA and 0.1% Triton X-100 in sterile PBS. Staining with anti-tubulin (Cell Signaling Technologies) was performed overnight at 4°C. Cells were stained with fluorescent phalloidin (Thermo Fisher Scientific, Waltham, MA, USA) or DAPI for 30 and 5 min, respectively. The following dilutions of reagents were used: anti-Tubulin: 1:200, Phalloidin-Red (F-actin): 1:1,000, and DAPI: 1:1,000. Alexa Fluor-conjugated secondary antibodies were used at a 1:2,000 dilutions. Stained slides were mounted with Vectashield mounting medium (Vector Laboratories, Burlingame, CA, USA), and images were taken with a 20× or 40× lenses. Data were analyzed and fluorescent intensities were calculated using Olympus software.

### Cytotoxicity Assays and *In Vivo* Tumor Clearance

Natural killer-mediated cytotoxicity was quantified using [51Cr]-labeled target cells at varied E:T ratio. Percent specific lysis was calculated using amounts of absolute, spontaneous, and experimental [51Cr]-release from target cells as previously described (50). For analysis of *in vivo* tumor clearance, RMA and RMA/S cells were labeled with 5 µM cell trace violet and CFSE (Thermo Fisher Scientific, Waltham, MA, USA), respectively, for 10 min and quenched with 10% FBS 1640 medium. Cells were mixed at a concentration of 10 × 10^6^ cells/ml of each in PBS, and 200 µl was injected retro-orbitally into each mouse. Spleens were collected 18 h later, and the fluorescence of single-cell suspensions was analyzed by flow cytometry. The ratio of RMA/S to RMA cells was calculated. Clearance of RMA/S was compared with the NK cell-depleted and IgG control groups. For *in vivo* NK cell depletion, NK1.1 (PK136) and IgG (Isotype control) were purchased from BioXcell (West Lebanon, NH, USA) and 200 µg was injected intraperitoneally into each mouse 24 h before tumor challenge.

### Cytokine and Chemokine Quantification

IL-2-cultured NK cells were activated with plate-bound anti-NKG2D (A10; eBioscience), anti-CD137 (17B5; eBioscience), anti-NK1.1 (PK136; eBioscience), anti-anti-Ly49D (4E5; BD Pharmingen), and anti-CD244 (2B4; Biolegend) mAbs at 2 μg/ml. Culture supernatants were analyzed after 18–21 h by Bioplex assay (Bio-Rad Laboratories, Hercules, CA, USA). Intracellular cytokines were quantified using established methodologies (51). Briefly, NK cells were activated with 2 µg/ml plate-bound anti-NKG2D (A10) mAb for 12 h in the presence of Brefeldin-A for the last 4 h of activation. For mTORC1 inhibition, NK cells were activated in the presence of 100 ng/ml rapamycin (MilliporeSigma, St Louis, MO, USA). After 8 h, IFN-γ was quantified in the culture supernatants by enzyme-linked immunosorbent assay. Where necessary, NK cells that had been cultured in IL-2 were treated with 1 ng/ml IL-12 and 10 ng/ml IL-18 (PreproTech, Rocky Hill, NJ, USA), and the supernatants were analyzed similarly.

### Western Blotting

Whole cell lysate (15–20 mg) were generated in RIPA buffer containing protease and phosphatase inhibitors as previously described (7) and resolved using 10% SDS-PAGE gels, transferred to PVDF membranes, blocked with 5% milk powder, and probed with indicated antibodies in sterile PBS containing 5% BSA overnight. After 1 h incubation with HRP-conjugated secondary antibodies, membranes were washed and signals were detected using SuperSignal West Pico or Dura Chemiluminescent Substrates (Thermo Scientific, Waltham, MA, USA). The fold changes in the S6 phosphorylation following NKG2D-mediated activation were calculated using Image J software (NIH, Bethesda, MD, USA) and compared. The band intensities of phospho-protein were normalized against the respective total protein. The fold changes in phosphorylation following 5, 20, or 60 min of activation were calculated using these normalized values. Antibodies and dilutions are as follows: anti-IQGAP1 (BD Biosciences), 1:1,000, anti-actin (Santa Cruz Biotechnology) 1:500, anti-tubulin, anti-phospho-Akt (Ser473), anti-phospho-Akt (Thr308), anti-Akt, anti-IκBα, anti-phospho-Erk1/2 (Thr202/Tyr204) anti-Erk1/2, anti-phospho-Jnk1/2 (Thr183/Tyr185), anti-Jnk1/2, anti-phospho-S6 (Ser235/236), anti-phospho-S6 (Ser240/244), and anti-S6 were all purchased from Cell Signaling Technologies and used at a 1:1,000 dilution. HRP-conjugated goat-anti-mouse or goat-anti-rabbit secondary antibodies (Bio-Rad Laboratories) were used at 1:2,000 for subsequent chemiluminescent detection.

### RT-qPCR and Microarray Analysis

For IFN-γ-encoding mRNA quantification, NK cells were activated for 6 h and processed for RNA extraction (RNeasy Mini Kit; QIAGEN). Next, cDNA was generated using the iScript cDNA synthesis kit (Bio-Rad Laboratories) according to the manufacturer’s instructions. Real-time PCR was performed by using a previously published SYBR green protocol with an ABI7900 HT thermal cycler. Transcript in each sample was assayed in triplicate, and the mean cycle threshold was used to calculate the x-fold change and control changes for each gene using Δ*C*_t_. The control (housekeeping) gene actb was used for global normalization in each experiment. Primer sequences for *Ifng* were 5′-GACTGTGATTGCGGGGTTGT-3′ (sense) and 5′-GGCCCGGAGTGTAGACATCT-3′ (anti-sense). For whole transcriptome analysis by microarray, NK cells were activated with 2.5 µg/ml anti-NKG2D antibody for 3 h and RNA was extracted as specified above. RNA collected from NK cells derived from four WT and four *Iqgap1*^−/−^ mice were pooled, and cDNA was generated and hybridized to the 430_2 microarray platform with the assistance of Martin Hessner’s laboratory (Max McGee National Research Center for Juvenile Diabetes, MCW). Quality Control and data analysis were completed using the TAC software (Affymetrix).

### Puromycin Incorporation Assay

IL-2-cultured NK cells were left unstimulated or activated for 4 h with 5 μg/mL anti-NKG2D mitogenic antibody (A10, eBioscience). Golgi-plug (1:1,000; BD Biosciences) and, where indicated, 50 μg/ml cycloheximide (CHX) was added to the cells for an additional hour. CHX is a potent inhibitor of cellular translation and was used as the negative control for this experiment. Next, 10 μg/ml Puromycin (MilliporeSigma), was added to the cells for 15 min, and cells were harvested, fixed, and permeabilized using the intracellular staining protocol as previously described (48). The cells were then incubated with the anti-puromycin antibody (12D10; MilliporeSigma) at a 1:1,000 dilution. After two washes, AF-488 goat-anti-mouse secondary antibody (Thermo Fisher) was added at 1/1,000 for 30 min at 4 C. Finally, cells were washed, and intracellular fluorescence was determined using the MACSQuant Flow cytometer, and the data were analyzed using FlowJo software. Positive events were determined based on gating NK cells (CD3ε^−^NK1.1^+^) that did not receive puromycin as the fluorescence-minus-one negative staining control.

### Mitochondrial Stress Test Assay

Natural killer cells were cultured in IL-2 for 7 days, washed, and placed in Seahorse XF base media. Mitochondrial function and glycolytic capacity were evaluated using the Mito Stress Test Kit and Glycolysis Stress Test Kit, respectively. These procedures were conducted according to the manufacturer’s instructions (Seahorse Biosciences, North Billerica, MA, USA) using The MCW Cancer Center Bioenergetics Shared Resource (MCW, Milwaukee, WI, USA).

### Statistical Analysis

Statistical analysis for each experiment is as indicated in the individual figure legends.

## Results

### Loss of IQGAP1 Results in Decreased Number of Splenic NK Cells

**Figure 1:**
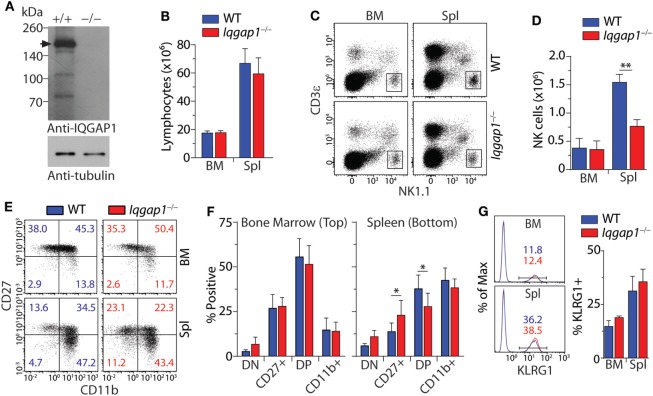
Characterization of natural killer (NK) cell development and maturation in *Iqgap1^−/−^* mice. **(A)** Evaluation of IQ domain-containing GTPase-activating protein 1 (IQGAP1) in wild-type (WT) and *Iqgap1^−/−^* NK cells by western blot. The black arrow indicates full-length IQGAP1 at approximately 190 kDa. Whole cell lysates were prepared from IL-2-cultured NK cells and α-tubulin serves as the loading control. **(B)** Flow cytometry was used to calculate lymphocyte numbers in the bone marrow (BM) and spleen based on FSC and SSC parameters. **(C)** Representative dot plots showing NK cell gate (CD3ε^−^NK1.1^+^) used to calculate total NK cell numbers in the BM and spleen as shown in panel **(D)**. **(E)** Representative dot plots of CD27 and CD11b expression on NK cells (CD3ε^−^NK1.1^+^) and **(F)** quantification of the DN (CD27^−^CD11b^−^), CD27^+^ (CD27^+^CD11b^−^), DP (CD27^+^CD11b^+^), and CD11b^+^ (CD27^−^CD11b^+^) populations were quantified in the BM and spleen. **(G)** Representative histograms are showing the percentage of KLRG1^+^ NK cells (CD3ε^−^NK1.1^+^) and quantification of these populations in the BM and spleen. Error bars represent SD using four to six mice in at least two independent experiments, *p < 0.05 and **p < 0.01 using two-tailed Student’s *t*-test.

To investigate the role of IQGAP1 in NK cell homeostasis, we analyzed their distinct developmental stages in a global gene knockout mouse (*Iqgap1^−/−^*). We confirmed the deletion of *Iqgap1* in NK cells from *Iqgap1^−/−^* mice by western blot. WT NK cells express the full-length IQGAP1 protein at 190 kDa as well as probable cleavage products (52), while *Iqgap1^−/−^* NK cells lack the full-length protein as well as any lower molecular weight cleavage products (Figure 1A). Although *Iqgap1^−/−^* mice maintain normal numbers of total lymphocytes (Figure 1B), the number of NK cells in the spleen of *Iqgap1^−/−^* mice was significantly reduced while total NK cell numbers were unaltered in the BM (Figures 1C,D). Analysis of NK cell maturation by differential surface expression of CD27 and CD11b (53, 54) revealed moderate alterations of CD27^+^ and CD27^+^CD11b^+^ NK cell populations in the spleens of *Iqgap1^−/−^* mice (Figures 1E,F). However, surface expression of the terminal maturation marker, KLRG1, was unaltered in *Iqgap1^−/−^* NK cells (Figure 1G). Ly49 receptor acquisition (Figures S1A,B in Supplementary Material), surface expression of NKRs (Figures S1C,D in Supplementary Material), and cell viability (Figure S1E in Supplementary Material) remained unchanged in *Iqgap1^−/−^* NK cells illustrating that NK cell development and maturation are largely intact in the absence of IQGAP1.

### IQGAP1 Regulates BM Egression and Motility of NK Cells

**Figure 2:**
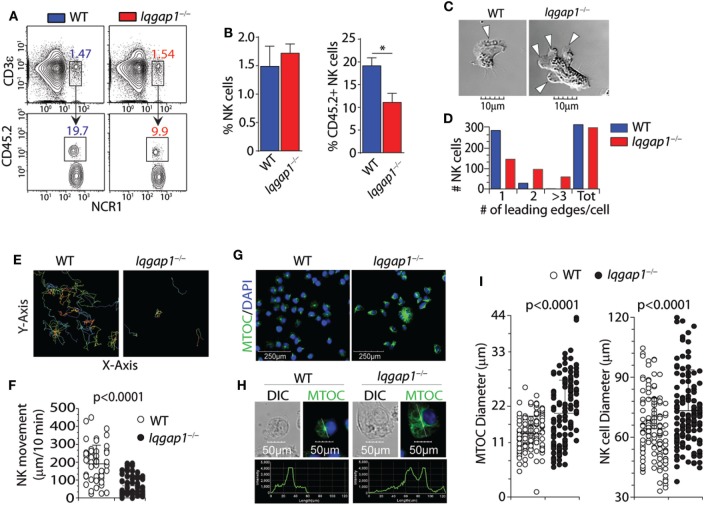
Bone marrow (BM) localization, cell polarization, and motility of *Iqgap1^−/−^* natural killer (NK) cells. **(A)** Representative dot plots and **(B)** quantified percentages of CD45.2-labeled NK cells (CD3ε^−^NCR1^+^) illustrating sinusoidal (CD45.2^+^) vs. parenchymal (CD45.2^−^) localization within the BM of wild-type (WT) and *Iqgap1^−/−^* mice. **(C)** Representative images of IL-2-cultured WT and *Iqgap1^−/−^* NK cells showing the formation of lamellipodia at the leading edge and **(D)** quantification of the total number of leading edges per cell in >250 NK cells. **(E)** Traces of IL-2-cultured WT and *Iqgap1^−/−^* NK cells with the quantified movement of these cells over time as shown in panel **(F)**. **(G)** Confocal microscopy of WT and *Iqgap1^−/−^* NK cells labeled with anti-tubulin antibody and counterstained with DAPI. **(H)** The intensity of tubulin fluorescence on a single-cell basis as well as quantification of microtubule organizing center (MTOC) and total NK cell diameter in 100 NK cells which are shown in **(I)**. Error bars represent SD **(B)** or SEM **(F,I)** using three to four mice in at least two independent experiments, *p < 0.05 using two-tailed Student’s *t*-test.

Molecular mediators, including chemokines and sphingolipids, regulate NK cell development and are vital in facilitating BM egress (55). Given the reduction in splenic NK cells in *Iqgap1^−/−^* mice, we investigated the localization of NK cells within the BM compartment. We analyzed the parenchymal and sinusoidal distribution of NK cells in the BM by *in vivo* labeling of vascular-associated (sinusoidal) cells with a fluorochrome-labeled CD45.2 antibody (56). Mice were intravenously injected with an anti-CD45.2 antibody, sacrificed after 2 min, and their BM cells were analyzed, which allowed us to quantify the number of NK cells in the sinusoidal vs. parenchymal regions of the BM, an indicator of NK cell trafficking *in vivo* (Figure 2A). We found a marked reduction of CD45.2-labeled sinusoidal NK cells in *Iqgap1^−/−^* mice, indicating an impairment in their ability to traffic (Figure 2B). Given the well-known role of IQGAP1 in mediating cell migration and motility (57, 58), we evaluated *Iqgap1^−/−^* NK cells *in vitro*. Contractile actomyosin complex helps to form blebs, and actin polymerization is required for lamellipodia expansion to facilitate coordinated cellular migration (59). To define the role of IQGAP1 in the directional movement of NK cells, we quantified the number of leading edges per NK in IL-2 culture. The majority of the NK cells from WT mice exhibited directional movement with a single-leading edge (Figure 2C). However, NK cells from *Iqgap1^−/−^* mice displayed multiple blebs and quantification of the number of lamellipodia suggested impaired directional motility (Figure 2D). To quantify the motility, we cultured the NK cells in IL-2 and, using a multiphoton confocal microscope, performed live cell imaging in real-time (Figure 2E). As expected, we observed a significant reduction in overall motility of *Iqgap1^−/−^* NK cells in IL-2 culture (Figure 2F).

### Lack of IQGAP1 Results in Impaired MTOC Morphology

To further assess cytoskeletal components involved in cellular motility and migration, we utilized confocal microscopy to evaluate the formation and morphology of the MTOC. Repositioning of the MTOC is an essential component of cellular polarity and is essential for directional cell movement (60). Similarly, the directional translocation of MTOC from the perinuclear region to the proximity of the plasma membrane is central to the formation of the immunological synapse and directed degranulation in NK cells. Lack of IQGAP1 resulted in considerable changes to the structure and appearance of the MTOC. NK cells from the WT mice contained well-defined MTOC located above the perinuclear region (Figure 2G, left panel). However, NK cells from *Iqgap1^−/−^* mice showed an increase in MTOC length associated with an overall increase in cell diameter (Figure 2G, right panel). We then quantified the size of the MTOC using a reference line across the longitudinal length of individual NK cells. Figure 2H shows representative NK cells from WT and *Iqgap1^−/−^* mice. Quantification of MTOC size in 100 individual NK cells from each strain revealed that the lack of IQGAP1 did not affect the formation of the MTOC; however, the overall size and morphology of the MTOC were significantly increased in *Iqgap1^−/−^* NK cells (Figure 2I). Interestingly, the significant increase in MTOC diameter observed in NK cells lacking IQGAP1 was also associated with morphologically irregular microtubule filaments and an overall increase in the fluorescent intensity of α-tubulin suggesting altered microtubule dynamics in *Iqgap1^−/−^* NK cells. Therefore, these data demonstrate a critical role for IQGAP1 in maintaining proper cytoskeletal structures and subsequent motility in NK cells.

### IQGAP1 Is Required for Optimal NK Cell-Mediated Antitumor Responses *In Vivo*

**Figure 3:**
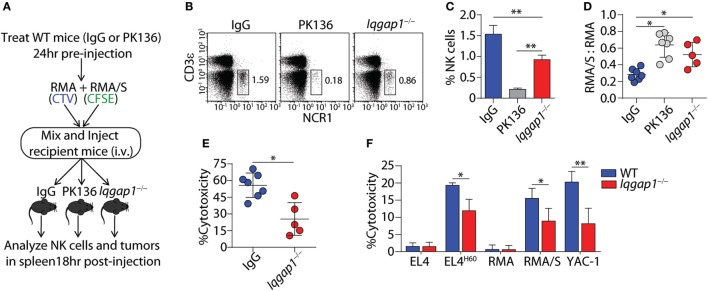
IQ domain-containing GTPase-activating protein 1 (IQGAP1) mediates the antitumor response of natural killer (NK) cells *in vivo*. **(A)** Diagram of “*missing-self*” tumor clearance model for *in vivo* evaluation of NK cell function. **(B)** Representative dot plots and **(C)** quantification of NK cell (CD3ε^−^NCR1^+^) percentages 18 h after tumor inoculation in wild-type (WT), isotype (IgG), or NK-depleted (PK136), as well as *Iqgap1^−/−^* mice. **(D)** Ratios of fluorescently labeled RMA/S to RMA cells collected from the spleens of antibody-treated WT or *Iqgap1^−/−^* mice 18-h post-inoculation and **(E)** *in vivo* cytotoxicity against RMA/S tumors in WT (IgG-treated) and *Iqgap1^−/−^* mice were calculated as described in the Materials and Methods section. **(F)** *Ex vivo* cytotoxic potential of WT and *Iqgap1^−/−^* NK cells was assessed by the standard 4-h ^51^Cr-release assay. Multiple tumor lines were co-cultured with splenic NK cells isolated from WT or *Iqgap1^−/−^* mice at an effector (E) to target (T) ratio of 10:1. Error bars represent SD using four to seven mice in at least two independent experiments, *p < 0.05 and **p < 0.01 using two-tailed Student’s *t*-test **(A–E)** or two-way ANOVA **(F)** accounting for multiple comparisons.

To ascertain the role of IQGAP1 in antitumor immunity, we employed the RMA-RMA/S tumor clearance model as an *in vivo* evaluation of NK cell function. Mice were depleted of NK cells by intra-peritoneal injection of the PK136 monoclonal antibody, 24 h before intravenous tumor inoculation, serve as the control for NK cell-mediated tumor clearance. Using this “*self*” vs. “*missing-self*” model (Figure 3A), *in vivo* cytotoxic potential of *Iqgap1^−/−^* NK cells was determined through changes in the ratio of RMA/S to RMA tumor cells recovered from the spleens of tumor-bearing mice when compared with WT mice as well as those sufficiently depleted of NK cells (Figures 3B,C). The ratio RMA/S to RMA cells recovered from the spleen of *Iqgap1^−/−^* mice was similar to that observed in NK cell-depleted mice, demonstrating an inability of *Iqgap1^−/−^* NK cells to mediate antitumor immunity (Figure 3D). Using the NK cell-depleted condition as a standard for no tumor clearance, we calculated the percent cytotoxicity of *Iqgap1^−/−^* NK cells and found that it was significantly reduced when compared with the IgG (WT) control (Figure 3E). To determine if the inability of *Iqgap1^−/−^* mice to clear RMA/S tumors was due to the reduction in peripheral NK cells or if the deficiency was on a per cell basis, we isolated splenic NK cells from WT and *Iqgap1^−/−^* mice and evaluated the cytotoxic potential of these cells against various targets using the standard 4-h ^51^Cr-release assay. We found that *Iqgap1^−/−^* NK cells were unable to mediate tumor killing to the level of WT controls (Figure 3F). This observation was consistent among multiple models that utilize distinct mechanisms of NK cell activation, implying a deficiency in the activation of critical molecular determinants of NK cell cytotoxic function in *Iqgap1^−/−^* NK cells.

### IL-2 Rescues Actin Polymerization and Cytotoxic Potential of *Iqgap1^−/−^* NK Cells

**Figure 4:**
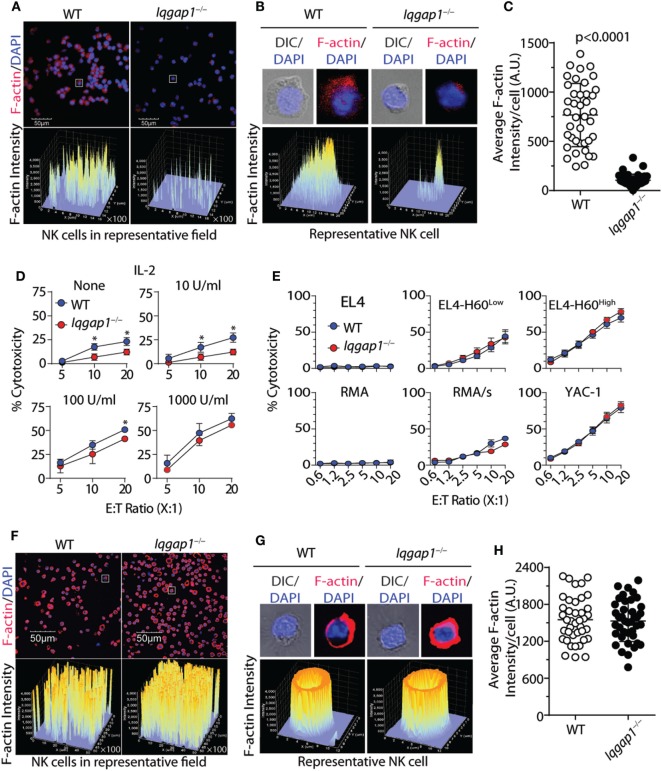
IL-2 rescues actin polymerization and cytotoxic potential of *Iqgap1^−/−^* natural killer (NK) cells. **(A)** Fluorescently labeled phalloidin was used to assess the accumulation of F-actin in splenic NK cells isolated from wild-type (WT) and *Iqgap1^−/−^* mice. These cells were counterstained with DAPI, and fluorescent intensity of phalloidin is shown for cell populations at 40× magnification. **(B)** Inset of representative cells isolated from WT and *Iqgap1^−/−^* mice indicated by the white box in panel **(A)**. **(C)** Quantified F-actin intensity in arbitrary units (A.U.) is shown for 40 cells selected from the field in panel **(A)**. **(D)** *Ex vivo* cytotoxic potential of WT and *Iqgap1^−/−^* NK cells was assessed by the standard 4-h ^51^Cr-release assay against YAC-1 targets at an effector to target ratio of 10:1 with increasing amounts of IL-2 added at the beginning of the assay. **(E)** Cytotoxicity of IL-2-cultured WT and *Iqgap1^−/−^* NK cells co-cultured with multiple tumor lines at the indicated effector to target ratios. **(F)** IL-2 cultured WT and *Iqgap1^−/−^* NK cells were treated as in panel **(A)**, and images were taken at 20× magnification. White boxes indicate WT and *Iqgap1^−/−^* NK cells used for single-cell inset in panel **(G)** where the fluorescent intensity of phalloidin is shown and **(H)** F-actin intensity was quantified as in panel **(C)**. Error bars represent SD **(C,H)** or SEM **(D,E)** using four to eight mice in at least two independent experiments, *p < 0.05 using two-tailed Student’s *t*-test.

Highly regulated reorganization of actin and tubulin-based cytoskeletal structures is a critical aspect of NK cell degranulation and cytotoxic function (61). Our observations demonstrate that IQGAP1 does indeed regulate cytoskeletal components, such as tubulin, in NK cells (Figure 2D). Given the known role for IQGAP1 in facilitating Wiskott–Aldrich syndrome protein (WASp)-dependent actin polymerization (17), and the potential impact that this may have on NK cell cytotoxicity (12, 62), we hypothesized that the reduced cytotoxic potential of IQGAP1-deficient NK cells was due to disrupted actin polymerization. Using fluorescent phalloidin as an indicator of F-actin, we evaluated actin polymerization in WT and *Iqgap1^−/−^* NK cells. A significant reduction in the fluorescent intensity of phalloidin in *Iqgap1^−/−^* NK cells imaged directly after isolation indicated impaired F-actin accumulation in these cells (Figures 4A–C). To determine if reduced actin polymerization in IQGAP1-deficient NK cells was the reason for the observed reduction in cytotoxic activity, we attempted to rescue NK cell-mediated cytotoxicity in *Iqgap1^−/−^* NK cells with the addition of recombinant IL-2 which is known to bypass WASp-dependent actin polymerization in NK cells (63). Interestingly, the addition of recombinant IL-2 during the ^51^Cr-release assay was able to rescue the ability of *Iqgap1^−/−^* NK cells to lyse YAC-1 targets in a dose-dependent manner (Figure 4D). IL-2-cultured *Iqgap1^−/−^* NK cells were also able to kill various tumor cells *in vitro* (Figure 4E). Importantly, this IL-2-mediated rescue in cytotoxic potential was associated with restored F-actin accumulation in *Iqgap1^−/−^* NK cells (Figures 4F–H). Therefore, these data demonstrate the ability of IQGAP1 to mediate actin polymerization required for optimal NK cell antitumor activity.

### Lack of IQGAP1 Impairs Cytokine Production in NKR-Stimulated NK Cells

**Figure 5:**
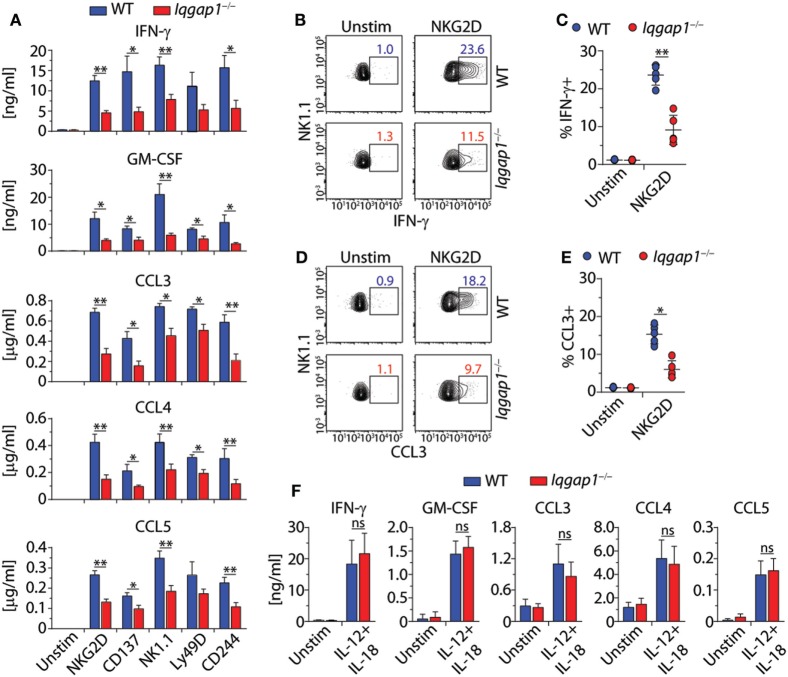
IQ domain-containing GTPase-activating protein 1 (IQGAP1) regulates inflammatory cytokine production in natural killer (NK) cells. **(A)** Cytokine (IFN-γ and GM-CSF) and chemokine (CCL3, CCL4, and CCL5) production by wild-type (WT) and *Iqgap1^−/−^* NK cells were assessed 18–21 h post-NKR stimulation (2 µg/ml) by BioPlex mouse cytokine assay. **(B,C)** Intracellular IFN-γ and **(D,E)** CCL3 accumulation was evaluated by flow cytometry 12-h post-NKG2D stimulation (2 µg/ml), and the data are quantified as percent positive NK cells (CD3ε^−^NK1.1^+^). **(F)** Cytokine and chemokine production by WT and *Iqgap1^−/−^* NK cells was assessed 18–21 after the addition of IL-12 (1 ng/ml) and IL-18 (10 ng/ml) by BioPlex mouse cytokine assay. Error bars represent SEM **(A,F)** or SD **(C,E)** using five to eight mice in at least two independent experiments, *p < 0.05 and **p < 0.01 using two-tailed Student’s *t*-test **(C,E)** or two-way ANOVA **(A,F)** accounting for multiple comparisons.

In addition to facilitating the functional reorganization of cytoskeletal components, IQGAP1 mediates multiple signaling pathways involved in a wide variety of cellular processes. Therefore, we hypothesized that loss of IQGAP1 would result in additional defects in NK cell function beyond cell-mediated cytotoxicity. To test this hypothesis, we investigated the ability of *Iqgap1^−/−^* NK cells to produce inflammatory cytokines. Evaluation of cytokine production in response to NKR activation revealed significant impairment in the ability of *Iqgap1^−/−^* NK cells to produce cytokines, such as IFN-γ and GM-CSF, as well as chemokines, such as CCL3, CCL4, and CCL5 (Figure 5A). Cytokines present in the supernatants of activated *Iqgap1^−/−^* NK cells were significantly reduced after stimulation through various NKRs including NKG2D, CD137, NK1.1, Ly49D, and CD244. Since IQGAP1 has previously been shown to regulate intracellular protein trafficking (64), we performed intracellular cytokine analysis by flow cytometry to determine if the defect in cytokine production was due to deficiencies in the exocytosis of cytokine-containing vesicles. To this end, we found a significant reduction in *Iqgap1^−/−^* NK cells containing IFN-γ (Figures 5B,C) and CCL3 (Figures 5D,E) in response to NKG2D stimulation. However, *Iqgap1^−/−^* NK cells were able to produce similar amounts of cytokines when stimulated with IL-12 and IL-18 when compared with WT controls (Figure 5F). These data suggest an NKR-specific role for IQGAP1 with regards to cytokine generation; therefore, we utilized NKG2D as a model receptor to further investigate this phenomenon.

### IQGAP1 Regulates Inflammatory Cytokine Production Through a Post-Transcriptional Mechanism

**Figure 6:**
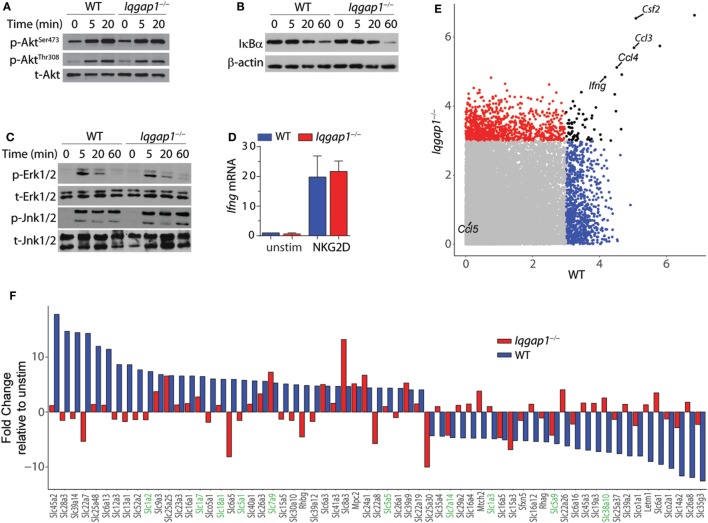
NKG2D signaling and induction of cytokine gene transcripts in *Iqgap1^−/−^* natural killer (NK) cells. **(A)** Phosphorylation of Akt at Ser^473^ and Thr^308^ was determined by western blot in NKG2D-stimulated wild-type (WT) and *Iqgap1^−/−^* NK cells at 20- and 60-min post-NKG2D activation and total Akt was used as the loading control **(B)** Degradation of IκBα is shown after activation with NKG2D in WT and *Iqgap1^−/−^* NK cells at indicated times post-activation with β-actin as the loading control. **(C)** Phosphorylation of mitogen-activated protein kinases, Erk1/2 (Thr^202^ and Tyr^204^), and Jnk1/2 (Thr^183^ and Tyr^185^), in WT and *Iqgap1^−/−^* NK cells are shown at indicated times post-NKG2D activation with total Erk1/2 and Jnk1/2 proteins serving loading controls. **(D)** Fold induction of *Ifng* transcript, relative to unstimulated WT NK cells, was determined by RT-qPCR 4-h post-NKG2D stimulation. **(E)** Microarray data represented by a scatter plot using a 3-Log2 fold change cutoff. NKG2D-induced cytokine transcripts are labeled. **(F)** Changes in the induction of solute carrier transcripts. Those that directly regulate amino acid transport are highlighted green. Error bars represent SD using four to five mice in at least two independent experiments **(A–D)** or four mice of each genotype pooled in one experiment **(E,F)**.

NKG2D stimulation results in the activation of critical signaling pathways that lead to cytokine gene transcription in NK cells (3). IQGAP1 regulates multiple kinase signaling programs known to be activated in response to NKG2D ligation, including Erk1/2 and Akt (14, 65). Therefore, we examined the requirement of IQGAP1 in NKG2D-mediated signaling in the context of cytokine production. Western blot analysis showed similar induction of Akt phosphorylation at both the Ser^473^ and Thr^308^ sites in *Iqgap1^−/−^* NK cells (Figure 6A). Furthermore, degradation of IκBα (Figure 6B) and the phosphorylation of MAP kinases, Erk1/2, and Jnk1/2 (Figure 6C) were also unaltered. Consistent with these observations, we found no defect in the induction of *Ifng* mRNA in *Iqgap1^−/−^* NK cells in response to NKG2D stimulation (Figure 6D).

To determine if IQGAP1 has a global effect on mRNA expression, we performed a genome-wide transcriptome analysis by microarray. Comparison of NKG2D-activated WT and *Iqgap1^−/−^* NK cells showed similar induction of many cytokine mRNAs including *Ifng, csf1, ccl3*, and *ccl4* (Figure 6E). Thus, the reduction in inflammatory cytokine generation by *Iqgap1^−/−^* NK cells appears to be regulated by a post-transcriptional mechanism. Interestingly, *Iqgap1^−/−^* NK cells showed notable deregulation in the mRNA expression of several solute carrier proteins (Figure 6F). These included amino acid transporters, such as the glutamine transporters, SLC1A2 and SLC1A7. Emerging evidence suggests a role for amino acid transporters as amino acid sensors capable of monitoring nutrient availability to control the activation of mTORC1, a master regulator of protein synthesis (66, 67). Although IQGAP1 has not been directly implicated in amino acid sensing, previous reports have demonstrated a functional interaction between IQGAP1 and mTORC1 (22, 46). Therefore, we next evaluated the contribution of mTORC1 activation to IFN-γ production and addressed mTORC1 activation in *Iqgap1^−/−^* NK cells.

### IQGAP1 Promotes NKG2D-Induced mTORC1 Activation in NK Cells

**Figure 7:**
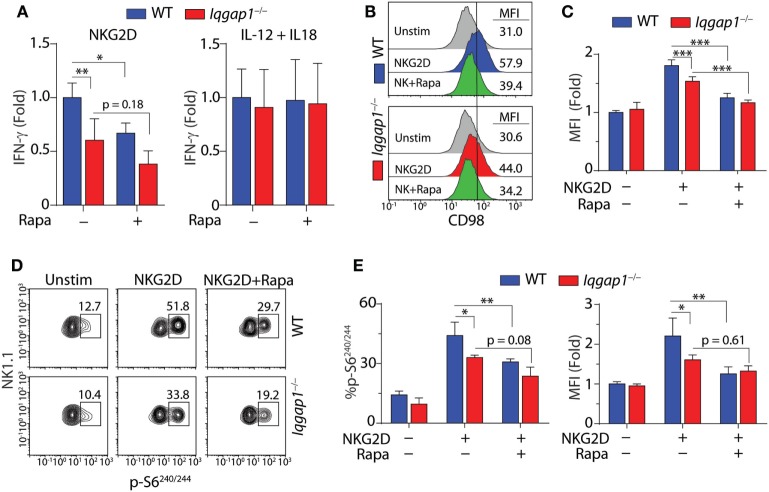
Activation of mechanistic target of rapamycin complex 1 (mTORC1) in NKG2D-stimulated natural killer (NK) cells. **(A)** Fold change in IFN-γ, relative to activated wild-type (WT) NK cells, was evaluated by ELISA in WT and *Iqgap1^−/−^* NK cells 8-h post-NKG2D or IL-12 and IL-18 stimulation with our without the addition of 100 nM rapamycin. **(B)** Upregulation of CD98 cell surface expression was evaluated by flow cytometry 8-h post-NKG2D activation in WT and *Iqgap1^−/−^* NK cells (CD3ε^−^NK1.1^+^). Histograms show representative MFI for each condition and quantified data relative to unstimulated WT NK cells is shown in panel **(C)**. Rapamycin (100 nM) was added during the 8-h stimulation as a negative control for mTORC1-mediated upregulation of CD98 surface expression. **(D)** Phosphorylation of S6 at Ser^240/244^ in WT and *Iqgap1^−/−^* NK cells was evaluated by intracellular flow cytometry 1-h post-NKG2D stimulation with or without 200 nM rapamycin to illustrate mTORC1-dependent S6 phosphorylation. Shown are representative contour plots with percent positive events within each gate and quantified data represented by percent phospho-S6 positive CD3ε^−^NK1.1^+^ NK cells, as well as fold change in MFI relative to unstimulated WT NK cells, is shown in panel **(E)**. Error bars represent SD using four mice in two independent experiments, *p < 0.05, **p < 0.01, and ***p < 0.001 using two-way ANOVA accounting for multiple comparisons.

To address the requirement of IQGAP1-mediated mTORC1 activation for IFN-γ production in NK cells, we inhibited mTORC1 with rapamycin (Figure 7A). Rapamycin treatment resulted in a significant decrease in IFN-γ production in NKG2D-stimulated WT NK cells; however, while rapamycin treatment also reduced IFN-γ production in *Iqgap1^−/−^* NK cells, the change was not statistically significant (Figure 7A). These data are consistent with a previous report demonstrating that mTOR is dispensable for IFN-γ production in response to IL-12 and IL-18, while IFN-γ production in response to NKR stimulation requires mTOR signaling (36). Since the reduction in IFN-γ generation in *Iqgap1^−/−^* NK cells mirrors that of rapamycin-treated WT NK cells when stimulated *via* NKG2D and *Iqgap1^−/−^* NK cells have no apparent defect in IFN-γ production in response to IL-12- and IL-18-mediated stimulation, we sought to further evaluate mTORC1 activation and signaling in response to NKG2D stimulation in *Iqgap1^−/−^* NK cells.

The surface expression of the amino acid transporter CD98 is positively regulated by mTORC1 activation (68, 69), and in NK cells, its induced expression is impaired by rapamycin treatment (38). As shown in Figures 7B,C, we found that NKG2D-induced surface expression of CD98 was significantly reduced in *Iqgap1^−/−^* NK cells. To further examine this defect, we investigated the mTORC1-mediated activation of specific signaling molecules in response to NKG2D stimulation. The ribosomal protein S6 is a component of the 40S ribosomal subunit and is phosphorylated at Ser^240/244^ by p70S6 kinase (S6K1), a direct target of mTORC1 (41). Using intracellular flow cytometry, we found a significant reduction in the phosphorylation of S6 at Ser^240/244^ following NKG2D stimulation in *Iqgap1^−/−^* NK cells (Figures 7D,E). Taken together, these results suggest a role for IQGAP1 in promoting NKG2D-mediated mTORC1 activation in NK cells.

### Phosphorylation of S6 and Global Protein Synthesis Are Reduced in *Iqgap1^−/−^* NK Cells

**Figure 8:**
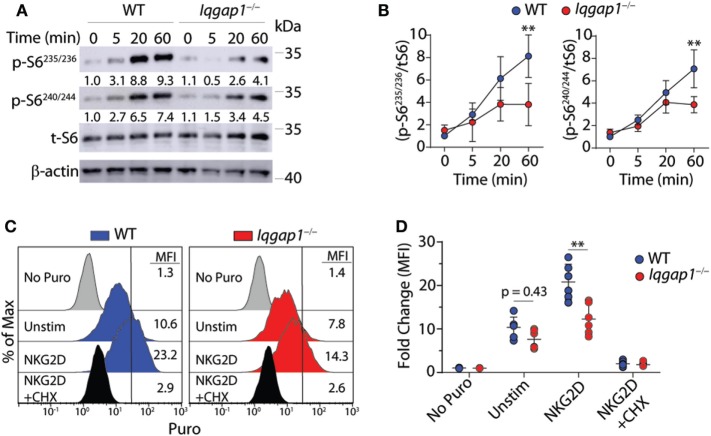
IQ domain-containing GTPase-activating protein 1 (IQGAP1) promotes sustained S6 activation and global protein synthesis in natural killer (NK) cells. **(A)** Phosphorylation of S6 at Ser^235/236^ and Ser^240/244^ was evaluated by western blot in IL-2 cultured wild-type (WT) and *Iqgap1^−/−^* NK cells at the indicated times post-NKG2D stimulation. Total S6 was used to calculate representative fold changes which are shown relative to unstimulated WT NK cells and quantification of four independent western blots is shown in **(B)** for both S6 phosphorylation sites. Global protein synthesis in WT and *Iqgap1^−/−^* NK cells was assessed by puromycin incorporation as described in the Materials and Methods section. **(C)** Representative histograms of puromycin incorporation gated from CD3ε^−^NK1.1^+^ NK cells are labeled with MFIs and **(D)** fold change in MFI was calculated relative to WT NK cells not treated with puromycin. Cycloheximide (CHX) was added during the experiment as a negative control for global protein synthesis. Error bars represent SD using four to six mice in at least two independent experiments, **p < 0.01 using two-way ANOVA accounting for multiple comparisons.

To further investigate the activation of the mTORC1 → S6K1 → S6 signaling axis in *Iqgap1^−/−^* NK cells, we conducted a time course experiment to examine S6 phosphorylation in response to NKG2D stimulation by western blot. As shown in Figures 8A,B, *Iqgap1^−/−^* NK cells were unable to sufficiently phosphorylate S6 at Ser^235/236^ as well as Ser^240/244^. Upon mitogenic stimulation, S6 is phosphorylated at Ser^235/236^ by P90S6K (RSK) through activation of the MAPK pathway; however, rapamycin-mediated inhibition of mTORC1 decreases these phosphorylation events (70–72), which suggests an exclusive role for mTORC1 in regulating S6 phosphorylation at multiple residues. Phosphorylation of S6 promotes its recruitment to the mRNA cap-binding complex and may play a key role in facilitating translation initiation (71), which suggests a potential role for IQGAP1 in mediating translation in activated NK cells. To evaluate global protein synthesis, we utilized a puromycin incorporation assay. Puromycin is a structural analog of aminoacyl tRNAs and is incorporated into the nascent polypeptide chain of an actively translating ribosome (73, 74). Thus, the amount of puromycin retained within a cell directly relates to the amount of protein synthesis occurring within that cell. Analysis of puromycin incorporation in *Iqgap1^−/−^* NK cells revealed a significant decrease in global protein synthesis in response to NKG2D stimulation (Figures 8C,D). Along with protein translation, mTORC1 also regulates metabolic reprogramming; therefore, we assessed the metabolism of *Iqgap1^−/−^* NK cells using the Seahorse stress test system (Figures S2A–D in Supplementary Material). The rates of extracellular acidification (ECAR) and oxygen consumption (OCR) were unaltered in *Iqgap1^−/−^* NK cells, suggesting that mTORC1 activity and the metabolic processes regulated by this complex are maintained in *Iqgap1^−/−^* NK cells in the absence of NKR stimulation. Since *Iqgap1^−/−^* NK cells were unable to sufficiently translate proteins in response to activation *via* NKG2D, our data suggest a role for IQGAP1 in regulating NKR-mediated cytokine translation through the activation of mTORC1.

## Discussion

In this study, we define multiple roles for IQGAP1 in regulating NK cell physiology. Despite having no apparent defects in development or maturation, *Iqgap1^−/−^* mice have significantly less splenic NK cells and altered NK cell distribution within the BM. This apparent deficiency in BM egress is associated with altered cell polarization and motility. However, it is possible that IQGAP1 plays a highly specific role in NK cell homeostasis within the BM. NK cell development occurs within the BM, and their movement between specific niches is a highly regulated process controlled by chemokine gradients (55). Furthermore, retention of NK cells within the BM is controlled by CXCL12–CXCR4 signaling. Indeed, IQGAP1 has been shown to regulate chemokine receptor signaling in immune cells. For instance, IQGAP1 interacts with CXCR2 in neutrophils (30) and promotes CXCR4 function and trafficking in Jurkat T cells (75). The role of IQGAP1 in these interactions may be of importance with regard to BM egression of NK cells; however, IQGAP1 can also directly regulate cell migration by regulating phosphoinositol signaling at the leading edge of migrating fibroblasts (57). Therefore, given the marked decrease in leading edge formation and cell motility *in vitro*, our data suggest that the alterations within the BM compartment are due to defects in conserved signaling mechanisms that are critical for leading-edge formation and cell migration.

We also observed a significant deficiency in the antitumor response of *Iqgap1^−/−^* NK cells. The inability of *Iqgap1^−/−^* mice to sufficiently clear RMA/S tumors *in vivo* demonstrates an apparent defect in the ability of *Iqgap1^−/−^* NK cells to mediate antitumor immunity in response to this “*missing-self*” tumor. The RMA tumor cell line is of C57BL/6 origin and maintains normal levels of MHC Class I surface expression. Therefore, RMA cells are recognized by NK cells as immunological “*self*” and are not targeted for lysis due to interactions between MHC Class I expressed on the target cell and inhibitory receptors on NK cells (76). Conversely, the RMA/S tumor line, derived from RMA, harbors a nonsense mutation in Tap2, a key peptide transporter and regulator of MHC Class I surface expression (77, 78). This mutation results in a marked reduction of MHC Class I surface expression and represents the immunological “*missing-self*.” In the absence of inhibitory signaling induced by MHC Class I expressed on the target cell, NK cells are able to efficiently lyse RMA/S tumors through the targeted release and delivery of granules containing the pore-forming enzyme, perforin, along with a specific class of serine proteases known as granzymes (79). Moreover, when isolated and co-cultured with various tumor cells, *Iqgap1^−/−^* NK cells have significantly reduced cytotoxic potential, which suggests a role for IQGAP1 in mediating NK cell cytotoxicity through a cell-intrinsic mechanism.

Specific F-actin reorganization mediated by actin-related protein-2/3 branching is required for the cytotoxic function of NK cells (80). Given that F-actin in isolated *Iqgap1^−/−^* NK cells is compromised, it is likely that IQGAP1 regulates NK cell cytotoxicity *via* actin polymerization. In line with this idea, the addition of IL-2 did indeed rescue the cytotoxic potential as well as the ability of *Iqgap1^−/−^* NK cells to accumulate F-actin. It is known that IQGAP1 stimulates actin polymerization through WASp (17) and WASp is required for cytotoxicity in uncultured NK cells (62). However, in a study using NK cells isolated from patients with Wiskott–Aldrich syndrome, Orange et al. demonstrated the ability of IL-2 to rescue both cytotoxicity and actin polymerization in WASp-deficient NK cells (63). Furthermore, this group showed that IL-2 was able to bypass WASp-dependent actin polymerization by activating the WASp-related actin reorganizer, WASp-family verprolin-homologous protein (WAVE) (63), and IQGAP1 does not mediate actin polymerization through WAVE (17). Therefore, it appears that IL-2 renders IQGAP1 dispensable for actin polymerization and, by extension, cytotoxicity in IL-2-conditioned NK cells.

Given the role of IQGAP1 in regulating multiple signaling pathways necessary for NK cell function, we investigated the ability of *Iqgap1^−/−^* NK cells to produce inflammatory cytokines. Our data show that *Iqgap1^−/−^* NK cells produce significantly less cytokines in response to NKR activation; however, *Iqgap1^−/−^* NK cells have no defect in cytokine production when activated with IL-12 and IL-18. Activation of NK cells *via* NKRs, such as NKG2D, and cytokines, such as IL-12 and IL-18, initiate distinct signaling programs in NK cells. For instance, IL-18 alone is not sufficient to stimulate IFN-γ production in NK cells but synergizes with IL-12 signaling (81). Therefore, a marked increase of IFN-γ production is expected when IL-18 is added in combination with IL-12 to activate NK cells. IL-12 signaling is propagated by the Janus-activated kinase/signal transducer and activator of transcription (STAT) pathway (81) and IL-18, a member of the IL-1 cytokine family, signals *via* the IL-18 receptor (IL-18R) complex (82). Specifically, STAT4 induction by IL-12 promotes IFN-γ production by enhancing *Ifng* gene transcription (81), while IL-18R signaling activates p38 MAPK which enhances *Ifng* transcript stability (83).

Although stimulation of NK cells *via* IL-12 and IL-18 results in substantial IFN-γ production, chemokine generation *via* this activation mechanism is not as robust compared with activation through NKRs. Differences in signaling mechanisms may be one potential explanation for this discrepancy. Distinct from IL-12 or IL-18, signaling events initiated in response to NKG2D stimulation are mediated through the adaptor molecules DNAX-activating protein of 10 kDa (DAP10) and DNAX-activating protein of 12 kDa (DAP12) *via* YXXM tyrosine-based and immunoreceptor tyrosine-based activation motifs (ITAMs), respectively (3). Upon NKG2D stimulation, the YXXM motif of DAP10 recruits and activates the p85 subunit of phosphoinositide 3-kinase, while the ITAM motif of DAP12 recruits ZAP70 and Syk to mediate NK cell activation *via* PLC-γ (84, 85). NGK2D signaling mediated through either DAP10 or DAP12 activates MAP kinases, Erk1/2 and P38, as well as Akt which results in the activation of critical transcription factors, such as AP-1 and NF-κB, that promote cytokine gene transcription (7). Therefore, IQGAP1 appears to regulate NKR-specific processes required for inflammatory cytokine generation.

Another essential difference between NKR and IL-12- and IL-18-induced IFN-γ generation is the requirement of the metabolic regulator, mTOR (36). In fact, inhibition of the critical metabolic processes, glycolysis, and oxidative phosphorylation, significantly reduces NKR-mediated IFN-γ generation, whereas IFN-γ production in response to IL-12 and IL-18 remains unaffected in NK cells (86). Therefore, we assessed these two critical metabolic processes in *Iqgap1^−/−^* NK cells using the Seahorse stress test system. Consistent with the observation that *Iqgap1^−/−^* NK cells have no defect in IL-2-induced proliferation in our culture system (data not shown), the rates of extracellular acidification and oxygen consumption were unaltered in *Iqgap1^−/−^* NK cells suggesting that mTORC1 activity and the metabolic processes regulated by this complex are maintained in *Iqgap1^−/−^* NK cells in the absence of NKR stimulation.

To further investigate this defect in IFN-γ production, we evaluated vital signaling molecules activated downstream of NKG2D and found no differences in NKG2D-induced stimulation of these pathways in *Iqgap1^−/−^* NK cells. Importantly, we also found no difference in NKG2D-induced *Ifng* gene transcription in *Iqgap1^−/−^* NK cells. We next performed a genome-wide transcriptome analysis by microarray, which showed similar induction of NKG2D-induced cytokine transcription in WT and *Iqgap1^−/−^* NK cells. However, the mRNAs of many solute carriers were differentially expressed in *Iqgap1^−/−^* NK cells in response to NKG2D stimulation. Solute carrier proteins play a crucial role in cellular responses to nutrient availability by regulating mTORC1 activation through amino acid sensing (67); therefore, we sought to investigate further mTORC1 activation in *Iqgap1^−/−^* NK cells stimulated *via* NKG2D.

Using the mTORC1 inhibitor, rapamycin, we confirmed the requirement for mTORC1 in NKG2D-induced IFN-γ production and, as expected, IFN-γ production in response to IL-12 + IL-18 was unaffected by rapamycin. Further investigation of mTORC1 activation by CD98 upregulation and S6 phosphorylation demonstrated a deficiency of mTORC1 activation in *Iqgap1^−/−^* NK cells stimulated *via* NKG2D. S6 is a well-known regulator of cellular translation (71) and, consistent with these previous observations, global protein synthesis in response to NKG2D stimulation was reduced in *Iqgap1^−/−^* NK cells. Along with protein translation, mTORC1 also regulates metabolic reprogramming required for optimal NK cell functions (36, 86), suggesting defects in cytokine production in IQGAP1-deficient NK cells could be a result of metabolic defects in addition to protein translation. Consistent with the observation that *Iqgap1^−/−^* NK cells have no defect in IL-2-induced proliferation in our culture system (data not shown), the rates of extracellular acidification and oxygen consumption were unaltered in *Iqgap1^−/−^* NK cells, suggesting that mTORC1 activity and the metabolic processes regulated by this complex are maintained in *Iqgap1^−/−^* NK cells in the absence of NKR stimulation. The molecular mechanism(s) by which IQGAP1 might mediate mTORC1-driven processes remains to be shown in NK cells; however, our observations suggest an essential role for IQGAP1-mediated mTORC1 activation in regulating protein translation in NKR-stimulated NK cells.

Our results demonstrate multiple roles for IQGAP1 in regulating NK cell effector responses. Although the role of IQGAP1 in facilitating cytoskeletal reorganization and cytotoxicity has been described in the human NK-like cell line, YTS (12), whether the function of IQGAP1 in regulating mTORC1 signaling is conserved between mice and humans was not addressed in this study and remains to be determined. With 98% amino acid homology between mice and humans (9), IQGAP1 is a highly conserved scaffold protein. Therefore, investigations into how IQGAP1 modulates NK cell effector functions may have clinical applications as recent advances in cellular immunotherapy have led to the utilization of NK cells as effector lymphocytes with anti-cancer properties in patients suffering from a wide range of malignancies (87). NK cell therapy has shown great promise as an anti-cancer therapeutic in clinical trials (88). However, an in-depth understanding of how NK cells function at the molecular level is required to further advance the therapeutic potential of NK cells. Cytokine release syndrome, induced by excessive immune cell activation, has been associated with the use of cellular immunotherapy for cancer treatment and clinical intervention is required to mitigate this potentially dangerous condition (89). However, targeted genetic or pharmacological manipulation of cellular products pre-transfusion may serve as a promising approach to reduce or eliminate many of the adverse effects associated with cellular immunotherapy. Multiple studies have concluded that, in NK cells, cytokine secretion and cytotoxicity are regulated differentially downstream of ITAM-containing receptors, such as NKG2D (7, 90, 91). Our findings demonstrate that IQGAP1 facilitates NK cell function at multiple levels and plays distinct roles in facilitating cytotoxicity vs. cytokine production. Therefore, the molecular mechanisms by which IQGAP1 differentially regulates NK cell function is of clinical significance and warrants further investigation for the development of next-generation NK cell-based therapeutics.

## Ethics Statement

All mice used in this study were utilized responsibly, and all protocols were approved by the institutional IACUC committee at the Medical College of Wisconsin (MCW), Milwaukee, WI, USA.

## Author Contributions

AA designed the study and performed experiments, data collection, analysis, and drafted the original manuscript. AT, ZG, JS, CY, NS, and KD participated in data collection and assisted in manuscript preparation. MT and SM conceived of the study, participated in its design, and performed data analysis and writing of the manuscript. All the authors have read and approved the final manuscript.

## Conflict of Interest Statement

The authors declare that this study was conducted in the absence of any commercial or financial relationships that could be construed as a potential conflict of interest.

## Supplementary Material

Figure S1 Phenotypic characterization of natural killer (NK) cells in *Iqgap1^−/−^* mice. **(A)** Representative histograms showing Ly49 receptor expression on NK cells (CD3ε^−^NK1.1^+^) and **(B)** quantified data from bone marrow (BM) and splenic NK cells in wild-type (WT) and *Iqgap1^−/−^* mice. **(C)** Representative NK cell activation receptor expression and **(D)** quantified data from BM and splenic NK cells in WT and *Iqgap1^−/−^* mice. **(E)** Cell death was assessed using Annexin V and propidium iodide in splenic NK cells from WT and *Iqgap1^−/−^* mice. Error bars represent SD with four mice from two independent experiments, **p* < 0.05 using two-way ANOVA accounting for multiple comparisons.

Figure S2 Metabolic characterization of *Iqgap1^−/−^* natural killer (NK) cells. Seahorse assay was used to measure ECAR and OCR rates in IL-2 cultured wild-type and *Iqgap1^−/−^* NK cells. **(A,B)** A glycolytic stress test was used to evaluate glycolytic parameters following the addition of glucose, oligomycin, and 2-DG. **(C,D)** A mitochondrial stress test was used to evaluate oxidative phosphorylation following the addition of oligomycin, FCCP, and antimycin A. Error bars represent SD using three mice from two independent experiments.
