# Supplementary figures and images for: IQ Domain-Containing GTPase-Activating Protein 1 Regulates Cytoskeletal Reorganization and Facilitates NKG2D-Mediated Mechanistic Target of Rapamycin Complex 1 Activation and Cytokine Gene Translation in Natural Killer Cells
Source: Front Immunol. 2018 May 28;9:1168. doi: 10.3389/fimmu.2018.01168 (PMC5985319; doi:10.3389/fimmu.2018.01168)

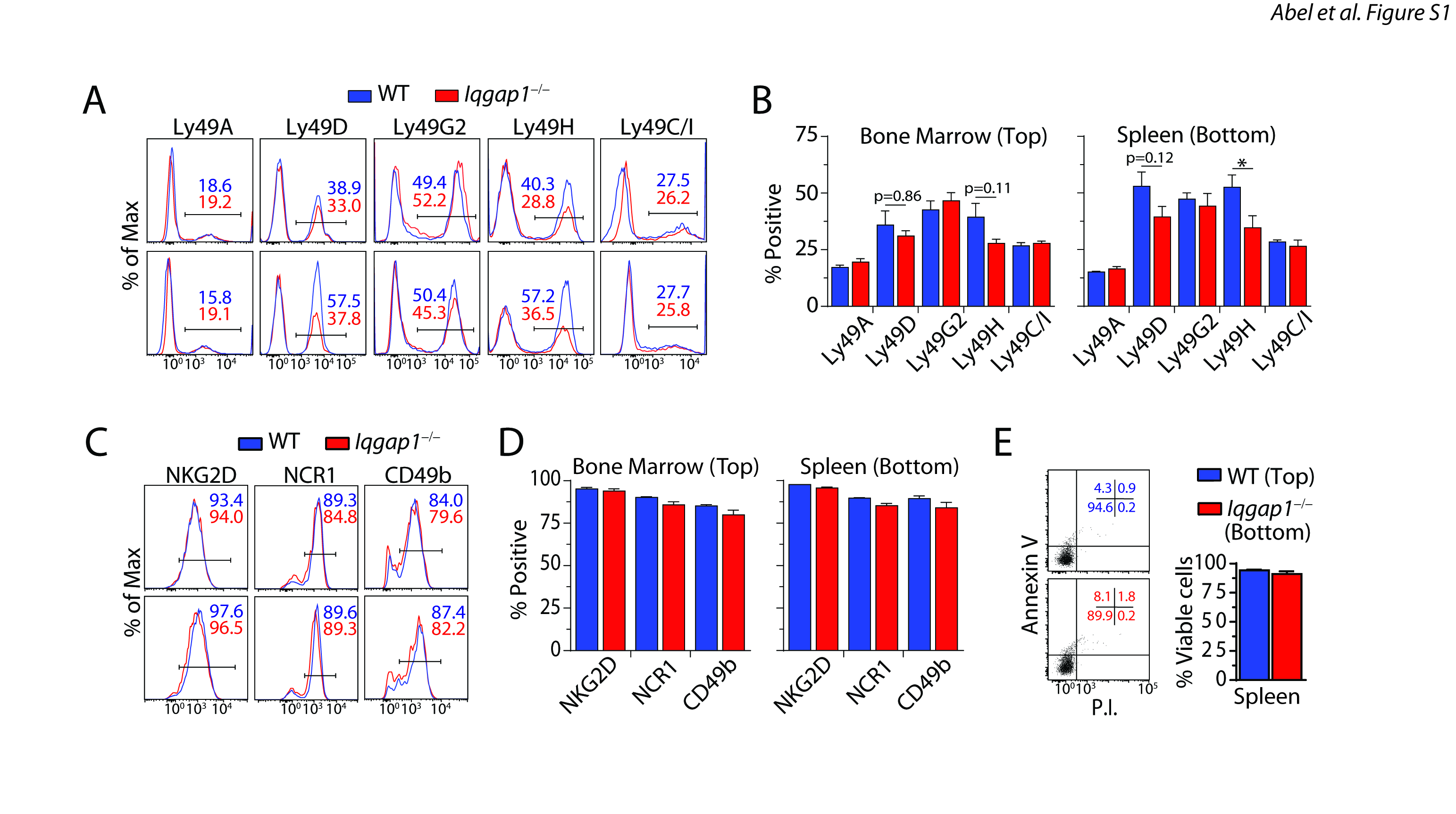

Supplement: Figure S1 — Phenotypic characterization of natural killer (NK) cells in Iqgap1−/− mice. (A) Representative histograms showing Ly49 receptor expression on NK cells (CD3ε−NK1.1+) and (B) quantified data from bone marrow (BM) and splenic NK cells in wild-type (WT) and Iqgap1−/− mice. (C) Representative NK cell activation receptor expression and (D) quantified data from BM and splenic NK cells in WT and Iqgap1−/− mice. (E) Cell death was assessed using Annexin V and propidium iodide in splenic NK cells from WT and Iqgap1−/− mice. Error bars represent SD with four mice from two independent experiments, *p < 0.05 using two-way ANOVA accounting for multiple comparisons. [file image_1.tif]

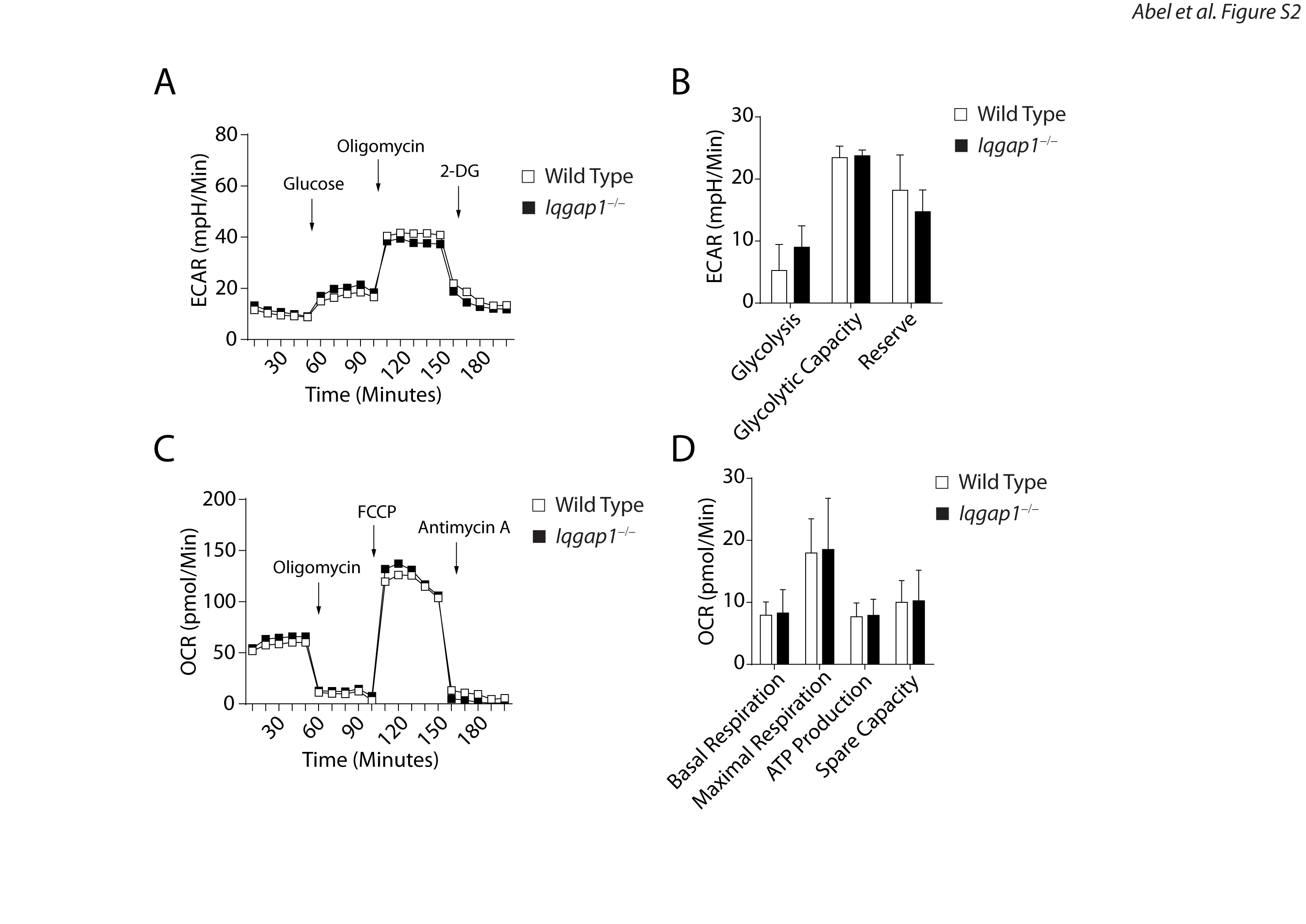

Supplement: Figure S2 — Metabolic characterization of Iqgap1−/− natural killer (NK) cells. Seahorse assay was used to measure ECAR and OCR rates in IL-2 cultured wild-type and Iqgap1−/− NK cells. (A,B) A glycolytic stress test was used to evaluate glycolytic parameters following the addition of glucose, oligomycin, and 2-DG. (C,D) A mitochondrial stress test was used to evaluate oxidative phosphorylation following the addition of oligomycin, FCCP, and antimycin A. Error bars represent SD using three mice from two independent experiments. [file image_2.tif]
